# First record of the invasive mosquito species *Aedes koreicus* (Diptera, Culicidae) in the Republic of Kazakhstan

**DOI:** 10.1051/parasite/2021050

**Published:** 2021-06-18

**Authors:** Yulia V. Andreeva, Natalia V. Khrabrova, Svetlana S. Alekseeva, Gulnar M. Abylkassymova, Anastasia V. Simakova, Anuarbek K. Sibataev

**Affiliations:** 1 Tomsk State University Lenin Street 36 634050 Tomsk Russia; 2 Institute of General Genetics and Cytology Al-Farabi Street 75 050060 Almaty Kazakhstan

**Keywords:** *Aedes koreicus*, Kazakhstan, Invasive mosquito species, Distribution

## Abstract

The natural distribution range of *Aedes koreicus* is Korea, China, Japan, and the Russian Far East. Since 2008, this species has been recorded as an invasive species in some European countries (Belgium, European Russia, Germany, Hungary, Italy, Slovenia, and Switzerland). The invasive mosquito species *Ae. koreicus* is reported from the Republic of Kazakhstan for the first time. Its morphological identification was confirmed by molecular-genetic analyses of ND4 sequences using specific primers. *Aedes koreicus* larvae were found in an artificial water reservoir together with the larvae of *Culiseta longiareolata* and *Culex pipiens* s.l. *Aedes koreicus* successfully overwintered in Almaty at low winter temperatures in 2018–2019. This suggests that the *Ae. koreicus* acclimation capacity is greater than it has been considered until now. We assume that *Ae. koreicus* will spread over the west and south of the Republic of Kazakhstan and territories of Kyrgyzstan and Uzbekistan Republics bordering the Almaty region.

## Abstract (fr)

L’aire de répartition naturelle d’*Aedes koreicus* est la Corée, la Chine, le Japon et l’Extrême-Orient russe. Depuis 2008, cette espèce a été signalée comme espèce envahissante dans certains pays européens (Belgique, Russie européenne, Allemagne, Hongrie, Italie, Slovénie et Suisse). L’espèce de moustique envahissante *Ae. koreicus* est signalée ici pour la première fois en République du Kazakhstan. Son identification morphologique a été confirmée par des analyses de génétique moléculaire de la séquence ND4 à l’aide d’amorces spécifiques. Des larves d’*Aedes koreicus* ont été trouvées dans un réservoir d’eau artificiel avec des larves de *Culiseta longiareolata* et *Culex pipiens* s.l. *Aedes koreicus* a hiverné avec succès à Almaty à de basses températures hivernales en 2018-2019. Cela suggère que la capacité d’acclimatation d’*Ae. koreicus* est supérieure à ce qui avait été envisagé jusqu’à présent. Nous supposons qu’*Ae. koreicus* s’étendra sur l’ouest et le sud de la République du Kazakhstan et les territoires du Kirghizistan et des républiques d’Ouzbékistan bordant la région d’Almaty.

## Introduction

*Aedes* mosquitoes are the most abundant mosquito species, and pose a health hazard for humans worldwide as well-known potential vectors of disease pathogens [35, 43]. During the last few decades, various mosquito species have spread expeditiously from their area of origin and colonised temperate zones [35]. In particular, the species *Aedes albopictus* Skuse, 1894 [45] and *Ae. japonicus* Theobald, 1901 [48] initially occupied East Asia areas. The species appeared in North America, Europe, and other regions as early as at the end of the 20th century [37]. Recent papers report recording of *Ae. aegypti* Linnaeus, 1762 [32] and *Ae. japonicus* in European countries, Russia included [1, 16, 22, 26, 27, 40, 41, 53]. Another species, *Ae. koreicus* Edwards, 1917 [17], has been introduced in some countries where it was not previously recorded. This species was recorded as an invasive species for the first time in 2008 in Belgium and later in Italy, Russia, Germany, Hungary, Slovenia and the Swiss–Italian border region [3, 6, 8, 18, 21, 27, 28, 31, 40, 46, 50, 52]. The natural distribution range of *Ae. koreicus* covers Korea, China, Japan, and the Russian Far East [24, 29]. This mosquito species is a potential vector of Japanese encephalitis, dirofilariasis, and Chikungunya virus [9].

One of the factors that contribute to the distribution of invasive species is an ever-increasing level of world travel and trade. Therefore, monitoring of the species composition of blood-sucking mosquitoes is required. Faunistic studies of the non-malaria mosquitoes in Central Asia and Kazakhstan have not been carried out for a long time. The most recent data on the species composition were published in 1970 by Dubitskii [14]. According to these data, the fauna of the *Aedes* mosquitoes in Kazakhstan comprises 38 species [14]. However, the *Systematic Catalog of Culicidae* reports only five *Aedes* species in Kazakhstan [20], namely, *Ae. gutzevichi* Dubitsky et Deshevykh, 1978 [15]*, Ae. stramineus* Dubitzky, 1970 [13]*, Ae. pulcritarsis* Róndani, 1872 [42]*, Ae. montchadskyi* Dubitsky, 1968 [12], and *Ae. kasachstanicus* Gutsevich, 1962 [23]. These considerable data discrepancies also require a faunistic study in the Republic of Kazakhstan. In addition, a sufficiently warm climate and the developing economy of the country are favourable factors for the introduction and establishment of invasive mosquito species.

## Materials and methods

Mosquito larvae were collected in September 2018 and May 2019 in Almaty, Republic of Kazakhstan (Table 1). The larvae were fixed in 96% ethanol. Late instar larvae were morphologically identified to species level according to the keys by Gutsevich et al. [24] and Tanaka et al. [47] using stereomicroscopes. Total DNA was extracted from individual mosquitoes (*n* = 12) using a GeneJet Genomic DNA purification kit (Thermo). A molecular assay based on nicotinamide adenine dinucleotide dehydrogenase subunit 4 (ND4) sequences was used for the identification of *Ae. koreicus* using multiplex PCR with the primers N4J-8502D (F) and N4N-8944D (R) [19], and ND4korF [7].

**Table 1:** Ratio of species in the collected samples of mosquito larvae.

	*Aedes koreicus*	*Culiseta longiareolata*	*Culex pipiens*
Almaty; 43°15′ N 76°58′ E September 19, 2018	75.3 ± 2.3%	21.0 ± 2.2%	3.7 ± 1.0%
	*n* = 262	*n* = 73	*n* = 13
Almaty; May 16, 2019	100%	0	0
	*n* = 51		

## Results

The larvae that we identified as *Ae. koreicus* according to morphological characteristics were found in September 2018 at Almaty zoo. Beside *Ae. koreicus*, this collection of larvae also contained larvae of *Culiseta longiareolata* and *Culex pipiens*. The collection of larvae sampled from the same artificial water reservoir (a bathtub) at the zoo in spring 2019 revealed only *Ae. koreicus* specimens (Table 1).

In its morphology, *Ae. koreicus* is similar to *Ae. japonicus,* both species display intraspecific alterations and, therefore, show overlapping morphological characteristics [36, 47]. Larvae from Kazakhstan show the same discriminating characteristics described by Tanaka et al. [47], which distinguish them from *Ae. japonicus*. The frontal setae of *Ae. koreicus* fourth instar larvae are located on the anterior margin of frontoclypeus. The abdominal segment VIII comb comprises 30–72 (54) wide paddle-shaped scales lacking a main spine. Pecten teeth evenly spaced, close to each other unlike *Ae. japonicus*, which has the most distal pecten teeth (one to four) detached, widely spaced, well developed, and forming a sharper corner to the siphon longitudinal axis [24, 47]. This siphon description of *Aedes koreicus* is well illustrated in Versteirt et al. [51]. We also identified *Ae. koreicus* species using a molecular approach. All specimens of *Ae. koreicus* displayed a specific band of 283 bp as well as the expected 465-bp band, common for other species.

## Discussion

The probability of an invasive species to colonise a new area depends on the climate characteristics of the region, availability of suitable aquatic habitats, and presence of suitable hosts [25, 34, 35, 38, 39, 49]. The knowledge of these specific features is of special significance for prediction of their future distribution. Presumably, the expansion of invasive species to new geographic regions is associated with their acclimation capacity at different developmental stages [5].

*Aedes koreicus* is well adapted to urban settlements [24, 38, 47]. We found *Ae. koreicus* larvae in a bathtub at the Almaty zoo. This might suggest that female *Ae. koreicus* mosquitoes can feed on both human and animal blood at the zoo. Gutsevich et al. [24] assumed that *Ae. koreicus* feed not only on humans, but also on cattle. The Italian team who discovered *Ae. koreicus* larvae in forest water bodies far from any settlements also inferred that females feed on animal blood [39]. So far, there are three species of mammals that were identified as hosts of *Ae. koreicus*: *Homo sapiens*, *Canis lupus* (may correspond to domestic animals) [39], and *Bos taurus* [49].

One of the major factors affecting the settlement and spread of invasive species is competition with native species [28, 35, 38]. *Aedes koreicus* was discovered in the region where the local mosquito species (for example, *Cs. longiareolata* and *Cx. pipiens* s.l.) also prefer artificial water reservoirs for their development. Based on preliminary data, we also assume that the ratio of these species changes throughout the year. *Aedes koreicus* was the first to appear in the collection of larvae at the beginning of the year in Almaty, while *Cs. longiareolata* and *Cx. pipiens* s.l. were found in the same water reservoir in September in addition to *Ae. koreicus*. The last two species also occur together with *Ae. koreicus* in Sochi (Russia) and in Germany [6, 40]. The larvae of *Cs. longiareolata* are predators [44], and they can compete for territory and food resources, but *Ae. koreicus* mosquitoes display a good acclimation capacity to a moderate climate; in particular, they produce cold-tolerant eggs, and the larvae hatch from these eggs during snow melting [29, 47]. Therefore, the tolerance of *Ae. koreicus* mosquitoes to lower temperatures allows them to start their development earlier and more massively as compared with their competitors, which hatch later.

*Aedes koreicus* occurs in countries with different climatic conditions. For the climate classification of the countries, we used the Köppen-Geiger world map, which was updated by Beck et al. [4]. According to the map, there is a dry/continental climate with warm (Dwb) and hot (Dwa) summers in North China, and a humid/temperate climate with warm summers in Belgium and parts of Germany. In north-eastern Italy, the climate is humid/temperate with hot summers, and in the Swiss-Italian border region, the climate is humid/continental with warm summers (Dfb climatic class), like in other European countries where *Ae. koreicus* has been found. In Almaty, the climate is temperate with hot summers. The climate is classified as Dfa according to the Köppen-Geiger system. *Aedes koreicus* type-locality (Korea) [47] has climatic conditions (Dfa, Dwa) similar to the Almaty region.

**Figure 1:**
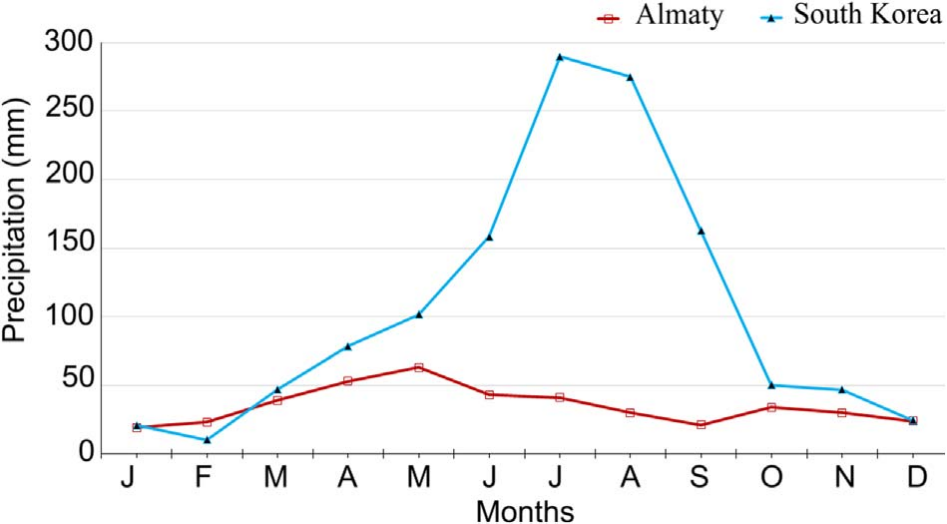
Comparison of the cumulative monthly precipitation (mm) at Almaty (Kazakhstan) and South Korea over 20 years.

**Figure 2:**
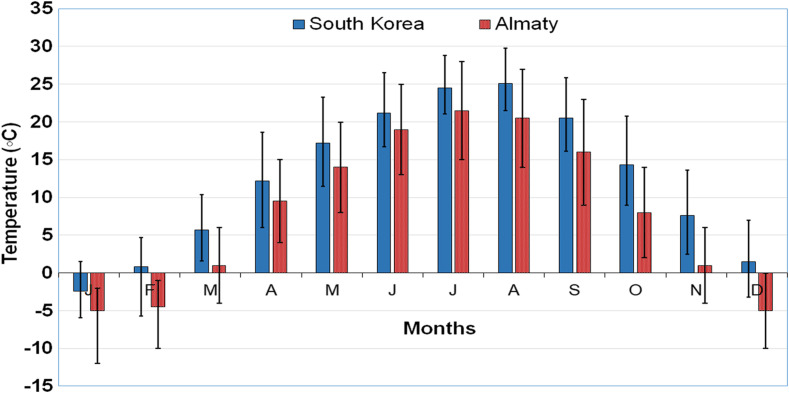
Comparison of the mean monthly temperatures at Almaty (Kazakhstan) and South Korea over 20 years. Vertical coloured lines in the upper part denote mean maximum temperatures (°C) over the considered period and in the lower part, mean minimum temperatures (°C).

We analysed the potential and prospects of expansion of the new species by comparing the data on temperature and humidity in Almaty [10, 11] and South Korea [30]. According to the data over the last 20 years, precipitation in Almaty is significantly lower as compared with South Korea (Fig. 1). However, lower precipitation is not likely to be a limiting factor for the development of *Ae. koreicus* in Almaty since this species feels quite comfortable in both natural and artificial aquatic habitats. As for the temperature, the average temperature is higher in Korea, with temperatures below zero occurring only in January. In Almaty, the minimum temperature in January over 20 years of observation exceeds –10 °C, while temperatures below zero can be observed during 3 winter months (December–February; Fig. 2).

**Figure 3:**
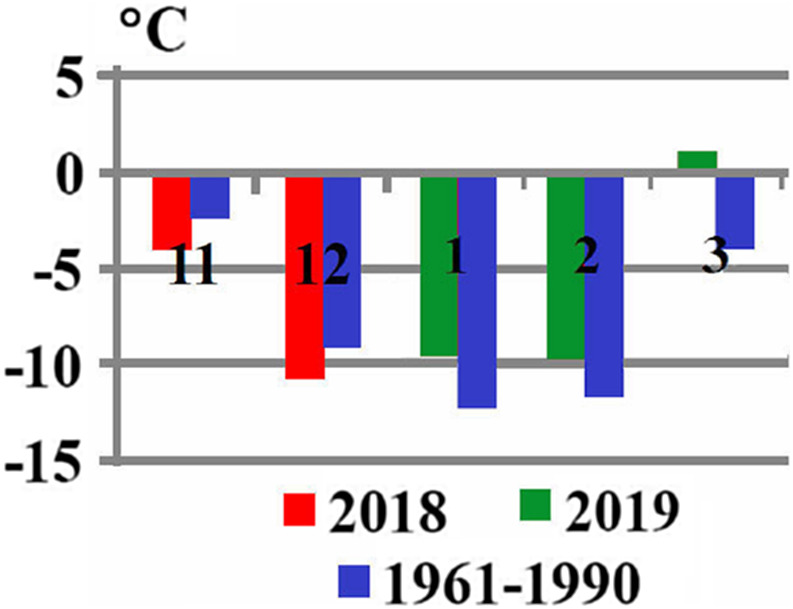
Comparison of average monthly temperatures of the low temperature season (November–March) at Almaty (Kazakhstan) in 2018–2019 and 1961–1990. Vertical coloured lines in the upper part denote mean maximum temperatures (°C) over the considered period and in the lower part, mean minimum temperatures (°C). The numbers indicate months: November (11), December (12), January (1), February (2), March (3).

Marini et al. [33] studied the effect of temperature on the *Ae. koreicus* bionomics and population dynamics. They showed in laboratory experiments that the most favourable temperature range for *Ae. koreicus* is 23–28 °C. Higher temperatures increase the pupal and adult lethality rates and prevent the female gonotrophic cycle, while lower temperatures, especially below 10 °C, slow down the development of immature stages and decrease the viability of eggs [33]. However, several studies demonstrate that *Ae. koreicus* is able to survive European winters [40, 50]. Our observation shows that *Ae. koreicus* successfully overwintered in Almaty with its low winter temperatures in 2018–2019 (Fig. 3). Thus, we assume that the *Ae. koreicus* acclimation capacity is greater than it has been considered until now, and this species is able to survive at rather low winter temperatures. In addition, the average annual and seasonal ground air temperatures in Kazakhstan have been observed to gradually increase over the last decade. The average annual air temperature in 1976–2018 increased by 0.31 °C every 10 years. The highest rate of temperature increase could be observed in spring (0.59 °C over 10 years) and in winter, it was the lowest (0.11 °C over 10 years). The temperature increased more rapidly in the western regions of Kazakhstan (from 0.24 to 0.60 °C over 10 years) and slower in the northern and northeastern regions (from 0.10 to 0.43 °C over 10 years), as well as in the mountain regions in the south (from 0.11 to 0.21 °C). The number of days with the average daily air temperature of 10 °C or higher increased by 3–5 days over 10 years and even by more than 5 days over 10 years in some southern regions [2]. Most likely, *Ae. koreicus* will spread over the territory of Kazakhstan westwards and southwards in the Almaty region, and it could cover the Kyzylorda, Turkestan, and Zhambyl regions and through the southwest of Aktyubinsk region to the West Kazakhstan, Atyrau, and Mangistau regions. The winter months in these regions are rather mild. The South and East of the Almaty region borders the Republics of Kyrgyzstan and Uzbekistan; most likely, *Ae. koreicus* as a species new to Central Asia will also spread to these territories. There are no recent faunistic studies; therefore, it can be assumed that other invasive species have already occupied their own niches in the blood-sucking mosquito fauna in the republics of Central Asia. The introduction route of *Ae. koreicus* is unknown, it may have occurred via international trade as Almaty is one of the largest industrial centers in Kazakhstan. The introduction of a new potential disease vector to Kazakhstan can be due to appropriate entrance points that emerged as a result of intense global trade and suitable environmental conditions. Regular monitoring of the mosquito fauna is necessary due to the development of transport routes, trade, and economic connections, which enhance the introduction of new species to the territories, where these species have not previously been recorded.

## Conflict of interest

The authors declare that they have no conflict of interest and they have observed all relevant ethical standards.
